# Modelling the Arctic wave-affected marginal ice zone: a comparison with ICESat-2 observations

**DOI:** 10.1098/rsta.2021.0262

**Published:** 2022-10-31

**Authors:** G. Boutin, T. Williams, C. Horvat, L. Brodeau

**Affiliations:** ^1^ Nansen Environmental and Remote Sensing Center, N-5007 Bergen, Norway; ^2^ Institute at Brown for Environment and Society, Brown University, Providence, RI 02912, USA; ^3^ CNRS, Institut de Géophysique de l’Environnement, 38 058 Grenoble, France

**Keywords:** sea ice, waves, modelling

## Abstract

We evaluate marginal ice zone (MIZ) extent in a wave–ice 25 km-resolution coupled model, compared with pan-Arctic wave-affected sea-ice regions derived from ICESat-2 altimetry over the period December 2018–May 2020. By using a definition of the MIZ based on the monthly maximum of the wave height, we suggest metrics to evaluate the model taking into account the sparse coverage of ICESat-2. The model produces MIZ extents comparable to observations, especially in winter. A sensitivity study highlights the need for strong wave attenuation in thick, compact ice but weaker attenuation as sea ice forms, as the model underestimates the MIZ extent in autumn. This underestimation may be due to limited wave growth in partially covered ice, overestimated sea-ice concentration or the absence of other processes affecting floe size. We discuss our results in the context of other definitions of the MIZ based on floe size and sea-ice concentration, as well as the potential impact of wave-induced fragmentation on ice dynamics, found to be minor at the climate scales investigated here.

This article is part of the theme issue ‘Theory, modelling and observations of marginal ice zone dynamics: multidisciplinary perspectives and outlooks’.

## 1. Introduction

The regions lying between open ocean and compact sea ice are collectively referred to as marginal ice zones (MIZs). In both hemispheres, MIZs are areas of particular importance for atmosphere–ocean–sea-ice processes (e.g. [1,2]), including interactions between waves and sea ice.

Sea ice inhibits wave generation and attenuates the waves propagating from open ocean. As a consequence, the presence of waves in sea ice has been suggested as a definition of the MIZ [3], since the distance over which waves propagate is approximately the extent over which open-ocean processes affect sea ice, mostly through the fracture of the ice by these waves. Once broken, sea ice is more sensitive to melt (lateral melt in particular [4–7]), less resistant to deformation [8] and more permissive to waves [9]. To properly assess the effect of waves on sea ice, we seek to properly estimate the extent over which waves are present in the ice and cause its fracture and to understand the key quantities that modulate this extent. Such an understanding is particularly crucial in a warming world, as reduced summer sea-ice extent and delayed autumn refreezing increases available fetch and permits the generation of more frequent and energetic waves in the Arctic [10]. Increasingly energetic waves also interact with thinner and more fragile Arctic sea ice [11,12], which is more susceptible to wave-induced fracture.

A number of dedicated modelling studies (e.g. [7,13–17]) and field campaigns (e.g. [18,19]) have recently attempted to characterize the impact of waves on sea ice. Modelling studies have mostly focused on two aspects: (i) wave attenuation in ice (e.g. [13,15,20,21]) and (ii) feedbacks of wave-induced ice break-up on sea ice and the ocean, such as enhanced lateral melting [7,16,17], lower ice strength [22,23] and sub-mesoscale eddy generation [5]. However, a major limitation of these modelling studies is that they are conducted at climate scales and therefore lack comparison against data that span large areas and long time periods (longer than a few weeks), which are not available via field campaigns. Indeed, most wave attenuation studies evaluate their models against only one or a few wave events (e.g. [14,15,20]), meaning that most wave attenuation parametrizations available in numerical models have not been evaluated in pan-Arctic/Antarctic simulations on climate time scales. Being able to evaluate wave attenuation over monthly time scales is all the more important given that dominant wave attenuation processes are known to strongly depend on ice properties such as the ice thickness, the floe size and the ice type [24–26], which vary strongly over time and between regions.

The lack of climate-scale evaluation of different modelling studies derives from an absence of observational datasets on wave–ice interactions (i.e. wave properties in ice and sea-ice floe size) covering large geographical areas (regional to global) over long time periods (more than a few days). Recently progress has been made by exploiting long-period moorings [27] and spaceborne radar or optical imagery [28–30]. A major advance has been made possible by satellite altimetry and particularly the recent launch of ICESat-2 (IS-2). The high (cm-scale) horizontal resolution of IS-2 measurements means that it can be used to directly measure waves in both open water and sea ice at long time scales and across the polar regions. IS-2 data have been used to estimate a wave-affected fraction (WAF) in both hemispheres, a quantity related to the extent of ice affected by waves [31]. Here, we will relate this WAF to quantities available as model output, as a method for understanding conclusions of previous modelling studies [23] investigating the effect of sea-ice break-up on sea-ice dynamics.

In this study, we evaluate the extent of ice affected by waves in the neXtSIM–WAVEWATCH III [32–34] coupled wave–sea-ice model [23] against the wave-affected marginal ice zone (wMIZ) estimated from IS-2 data in [31]. We introduce a method for evaluating standard wave and sea-ice model output against WAF values retrieved from IS-2. We assess the ability of our model to reproduce the wMIZ extent and discuss physical parameters affecting the results. We also discuss how the wMIZ extent compares with that based on floe size, which can also be observed from satellite altimetry [29,35], as well as the potential impact of wave-induced break-up on sea-ice dynamics.

## 2. Methods

### (a) ICESat-2 wave-in-ice data

The IS-2 dataset and retrieval method are described in detail in [31]. Briefly, IS-2 sea-ice height data are analysed for regions with high, wave-like variations. IS-2 tracks are divided into segments with a mean length of approximately 17 m. In [31] the segments were distinguished with a negative height anomaly from the others and considered to be wave affected. Then, the WAF was computed as the length of negative IS-2 heights divided by the length of all heights, for all segments binned monthly on a 100 km (or 25 km) polar stereographic grid over the period from October 2018 to the present. The main limitation of this method is that it only detects waves with large amplitudes (of at least 0.54 m) to ensure that a negative height anomaly is not due to sea-ice or sea-level variability. Even though validation against *in situ* data would be required to fully assess the uncertainties associated with wave detection using IS-2, recent studies [36,37] have demonstrated IS-2’s ability to capture wave signature in its data. The authors of [31] suggested that their method of filtering segments to determine whether they are wave affected is rather conservative and hence is more likely to exclude true wave observations than to include noise (due to ice roughness for instance). This statement is confirmed in [36], where spectral analysis was used to retrieve wave signatures in IS-2 data and the results were compared against those of [31]. Summer retrievals, however, should be considered carefully, as melt ponds can be mistaken for sea surface points, leading to overestimates of sea-level height and false positive segments in the wave detection algorithm.

**Figure 1.**
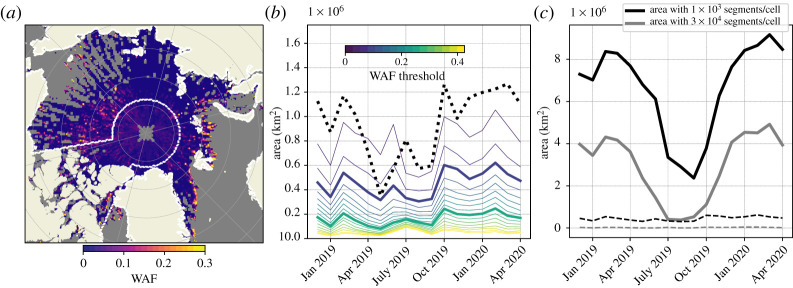
(*a*) Wave-affected fraction as defined in [31] in the Arctic for November 2019, using the 25 km dataset. The white contour around the pole and encompassing the Canadian Archipelago represents the part of the domain that is excluded for the model evaluation. (*b*) wMIZ extent evolution from the 25 km dataset using different WAF thresholds. The dotted line represents the wMIZ extent using the 100 km dataset and a WAF threshold of 0.075, as in [31]. (*c*) Evolution of total area covered by the dataset if we ignore cells with fewer than 1000 segments in a month (black solid line) or if we ignore cells with fewer than 30 000 segments in a month (grey solid line). The dashed lines represent the area with detected waves (WAF≥0.1) in both cases (cells with more than 1000 segments in black and those with more than 30 000 segments in grey). (Online version in colour.)

WAF values are reported only when more than 1000 segments are recorded in a given month in each bin, making the 25 km grid significantly noisier than the 100 km grid (figure 1*a* compared with figs S1 and S5 in the supplementary material of [31]). Yet given that waves rarely propagate more than 100 km into the ice cover, we choose to work with the 25 km dataset and address missing values below.

As in [31], we consider a grid cell to be ‘wave affected’ if the WAF exceeds a certain threshold, which we impose to limit the impact of noise. We qualitatively pick 0.1 as the threshold, as it eliminates a large part of the noise and shows a month-to-month variability of the wMIZ extent similar to that obtained using the 100 km version of the dataset and the threshold of 0.075 described in [31] (figure 1*b*). We use a higher threshold of 0.25 to delimit areas more confidently affected by waves. The observed wMIZ extent is relatively insensitive to further increases in this threshold. We also define a second high-confidence area by filtering out all cells with fewer than 30 000 segments observed monthly. This restriction reduces the total observed area by approximately 30% in the winter and 15% in the summer (figure 1*c*), and includes almost entirely observations of ‘no waves’ (WAF<0.1), as most of the cells that remain are found at latitudes higher than $75^{\circ }\;N$.

### (b) Coupled wave–ice model

We use the coupled wave–ice model developed in [23]. The wave model is WAVEWATCH III (hereafter referred to as WW3 [34]), and the sea-ice model is neXtSIM [33,38]. The model resolution is 25 km, the same as in the IS-2 dataset, and the model domain includes all the Arctic covered by the IS-2 dataset. We exclude closed seas (Hudson Bay and Baltic Sea) and the Canadian Archipelago from our analysis (see figure 1*a*), as we do not expect our model with a 25 km resolution to resolve wave growth and propagation in these areas.

The main difference between the model used in this study and that of [23] is that the sea-ice rheology in neXtSIM has been updated from the Maxwell elasto-brittle rheology [32] to the brittle Bingham–Maxwell (BBM) rheology [33]. The BBM rheology introduces a plastic threshold that prevents convergence unless ice is strongly damaged or undergoes strong external compressive stress. The potential impact of this change is discussed in §3e. The other differences from [23] are related to the model set-up and are described in detail in §S1 of the electronic supplementary material.

We define a reference simulation, denoted by REF, where we do not activate processes (lateral melting, link between damage and fragmentation; see [23]) related to the impact of waves on sea-ice properties, other than to update the floe size in the sea-ice model. This means that in REF, WW3 sends information about whether fragmentation occurs, as explained in [23], and neXtSIM updates the floe size. This floe size has no impact on sea-ice thermodynamics, drift or damage, and is sent back to WW3 where it is used to compute wave attenuation. Floe size in neXtSIM is only affected by wave-induced break-up and floe size growth mechanisms (refreezing and floe welding; see [23]).

We also run sensitivity experiments to investigate the effects of the values of the inelastic dissipation of wave energy through the B parameter as defined in [15] (eqn (20)), the roughness length $z_{0}$ associated with the contribution of turbulent friction to wave energy dissipation [39] (eqns (B3)–(B5)), the flexural strength of sea ice $\sigma_{\mathrm{flex}}$ [15] (eqn (12)), and the link between wave-induced fragmentation and damage [23]. Simulation names and the associated parameter values are summarized in table 1.

**Table 1.** Model parameters associated with each simulation described in §2b. In bold are the values that differ from the reference simulation.

name	link frag. and damage	$z_{0}\;(m)$	$B\;({N\;m}^{2}\;s^{1/3})$	$\sigma_{\mathrm{flex}}\;(\mathrm{MPa})$
REF	no	$10^{-4}$	$3\times 10^{7}$	0.6
DMG	**yes**	$10^{-4}$	$3\times 10^{7}$	0.6
LFXST	**yes**	$10^{-4}$	$3\times 10^{7}$	**0.247**
NIDIS	**yes**	$10^{-2}$	**0**	0.6

### (c) Other datasets

To evaluate simulated wave heights in open water, we use a wave-height dataset focusing on the Arctic compiled by Heorton *et al*. [40]. It provides daily estimates of wave height using the CryoSat-2 altimeter. These estimates are binned daily and averaged on a 100 km polar stereographic grid covering 2011–2019. For this comparison, we average daily simulated wave heights interpolated to the coarser observational grid.

For evaluation of sea-ice properties, we follow the methods of [41] and use the observations described therein: CS2-SMOS sea-ice thickness [42], OSI-SAF SSMIS sea-ice concentration [43] and the low-resolution 48 h OSI-SAF sea-ice drift [44].

## 3. Results

### (a) Evaluation of modelled ocean wave height and sea-ice properties

We first examine open-ocean wave conditions, with the results presented in figure S1 of the electronic supplementary material. Overall the model reproduces the observed wave energies, in particular with low significant wave-height bias in key Arctic regions of interest such as the Greenland, Barents and Bering Seas (figure S1*b,c*, electronic supplementary material). Wave heights tend to be underestimated within the Arctic Basin, such as in the Kara, Beaufort, Chukchi and East Siberian Seas, which are only ice free (and therefore usable for this comparison) for a short part of the year (approximately June to October). Wave heights are generally quite low in these regions, even when they are ice free (figure S1a, electronic supplementary material).

We also evaluate bulk sea-ice properties against observations, with the results summarized in table S2 of the electronic supplementary material. We find good agreement between the model and observed drift, but near-uniform overestimation of sea-ice extent except from July to August 2019. Most of this consistent bias is found in the Greenland Sea, with sea ice extending too far east (by 1–2 grid cells) and more compact than in observations (see figure S2, electronic supplementary material). As a result, the model is likely to overestimate wave attenuation and underestimate the wMIZ extent in this region. Conversely, in summer, overestimation of wave height in ice may arise from an overestimation of the available fetch. Sea-ice thicknesses are underestimated, mainly in the central Arctic (not shown), where waves are not expected to be found, at least in winter.

### (b) Comparison of modelled wMIZ extent against ICESat-2

As described in §2a, we seek to define a model wMIZ that can be compared with the region where the IS-2 WAF is higher than 0.1. The WAF provides information about the frequency of detection of waves with amplitude higher than 0.54 m (1.08 m wave height). In [31] this threshold was estimated on average for the Arctic and Antarctic. It depends on local properties such as the variability of sea-ice thickness and sea level. Assuming a Rayleigh distribution of the wave height, which seems reasonable as wave spectra in ice are generally narrow, a sea state with a significant wave height of $H_{s}=1.5\;m$ should have approximately 37% of individual waves exceeding 1.08 m. This is a significant amount of waves that should be identified by IS-2 and should significantly increase the WAF value for a given grid cell [31]. Thus, a consistent modelled definition of the wMIZ is the ice-covered area (i.e. with sea-ice concentration higher than 15%) with values of $H_{s}$ higher than approximately 1.5 m at least once in the month.

This definition is simple, but the maximum $H_{s}$ may give an upper bound on the extent observed by ICESat-2 as the sparse coverage of the altimeter is unlikely to capture the maximum value of the wave height at any given location. To estimate the sensitivity of our results to this wMIZ definition, we also use a definition of the wMIZ that considers the 90th percentile of wave heights in a month, a more conservative estimate of the modelled wMIZ. We also consider the uncertainty associated with the modelled $H_{s}$, as well as the detection threshold. Assuming a wave attenuation rate of the order of $10^{-5}\;(m/m)$, which is an upper bound on measured in-ice attenuation rates (attenuation being stronger at the ice edge [21]), the wave height decrease in a 25 km grid cell should be of the order of 0.25 m. The uncertainties linked to the detection threshold are difficult to estimate in the absence of extensive evaluation of the WAF retrieval method, but we address this issue in two ways. First, we assume an uncertainty of approximately 10 cm in this threshold, mostly due to variations of what Horvat *et al*. [31] call ν (linked to local surface properties, it is 20 cm on average but can be as low as 10 cm as found in [31]), resulting in an approximately 20 cm uncertainty for the minimum wave height that can be detected. Adding this uncertainty to that of the modelled wave height and rounding it up, we compare the observed wMIZ to the area with a monthly maximum value of the modelled $H_{s}$ higher than 1.50±0.5 m. Second, given that recent studies have shown clear wave signals in the IS-2 data for wave heights as low as 0.50 m (fig. 3*e* in [37]), we define the upper bound of the wMIZ extent as the area with a monthly maximum of $H_{s}$ higher than 0.50 m. This area is affected by waves that could be detected using the method of [31], assuming very little ‘noise’ (e.g. ice roughness) in the data, and is used to give an idea of the sensitivity of the wMIZ extent to lower wave heights.

**Figure 2.**
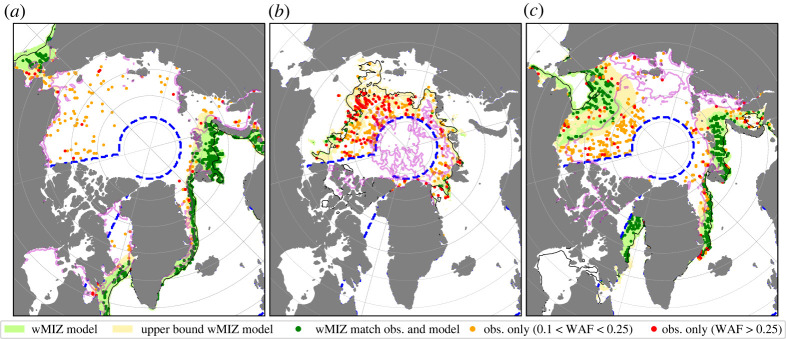
(*a*–*c*) Comparison of the wMIZ between the model (using REF and the maximum $H_{s}$ for each month) and IS-2, for the months of February (*a*), August (*b*) and November (*c*) 2019. The blue dashed line corresponds to the domain where we perform the quantitative analysis, ignoring the Canadian Archipelago, the Baltic Sea and Hudson Bay. The green and yellow shaded areas correspond to the modelled wMIZ using the criteria that the monthly maximum value of $H_{s}$ in ice is higher than 1.50 m and 0.50 m, respectively. Points with detected waves (WAF>0.1) within the wMIZ are in darker green. Red (orange) points show wave observations with WAF>0.25 (0.1<WAF<0.25) that are not encompassed by the modelled wMIZ. The thin solid black contour represents the area with an averaged total sea-ice concentration above 15%. The magenta contour represents the area with an averaged sea-ice concentration above 80%, ignoring newly formed ice in the model (see §A.2 of [38] for its definition). (Online version in colour.)

Maps of 2019 wMIZ extents are shown in figure 2, representing model behaviour for winter, summer and autumn. The modelled wMIZ extent in winter (figure 2*a*) captures most of the observed locations with a WAF exceeding 0.1 in the vicinity of the ice edge, though a number of points with high WAF values, over 0.25, are not captured farther in the ice pack of the Arctic Basin. A similar pattern holds in April and May, though there are fewer observations of waves close to the ice edge and more of them in pack ice not captured by the model (not shown). From June to August (figure 2*b*), the model fails to capture most of the observations of waves. This means either that the model underestimates wave heights in ice or that the data are very noisy in this period, as suggested in [31]. In November, the model performs well on the Atlantic sector, but the wMIZ extent in the Beaufort and Chukchi Seas is still underestimated, even when using the upper bound of the wMIZ extent.

**Figure 3.**
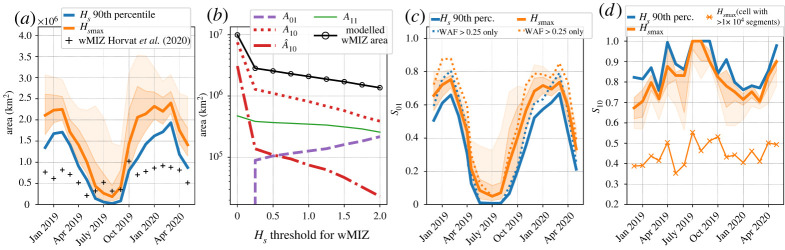
(*a*) wMIZ extent evolution depending on the definition of the wMIZ. The black line represents the wMIZ extent from IS-2 computed from the 100 km dataset as in [31]. The darker orange shaded area illustrates the sensitivity of the wMIZ extent for the maximum value of $H_{s}$ being 1 m (upper bound) and 2 m (lower bound). The lighter orange shaded area illustrates this sensitivity for a maximum value of $H_{s}$ as low as 0.5 m. (*b*) Extent of model match ($A_{11}$) and mismatch ($A_{10}$, $A_{01}$ and ${\hat{A}}_{10}$; see their definitions in §3b) with observations and MIZ area for different $H_{s}$ values used as a threshold for the wMIZ definition using the 90th percentile of $H_{s}$ for each month. For the computation of $A_{10}$ and ${\hat{A}}_{10}$, i.e. areas with waves in the model but not in observations, model cells are sampled only if IS-2 observations are available (i.e. no missing values in IS-2 dataset). (*c*) Time evolution of $S_{01}$, the fraction of the wave-affected area as observed from IS-2 that is captured by the modelled wMIZ using maximum $H_{s}$ (orange) and the 90th percentile of $H_{s}$ (blue), both with an $H_{s}$ threshold of 1.5 m. The upper and lower bounds of the orange shaded areas are the same as for (*a*). Dotted lines correspond to the same quantities but when only the high-confidence wave-affected area (WAF>0.25) is considered in $S_{01}$. (*d*) Same as (*c*) but this time for $S_{10}$, the percentage of the modelled wMIZ surface area that matches the observed wMIZ area from IS-2. The upper and lower bounds of the darker orange shaded area are reversed compared to (*b*). The lighter orange shaded area still represents the sensitivity using a maximum value of $H_{s}$ as low as 0.5 m, and $S_{10}$ is computed using ${\hat{A}}_{10}$. The thin solid line with crosses represents the evolution of $S_{10}$, with ${\hat{A}}_{10}$ computed using cells with more than 10 000, instead of 30 000, segments. (Online version in colour.)

The evolution of integrated wMIZ extent is shown in figure 3*a* (in orange) along with the wMIZ extent estimated using the binned 100 km IS-2 dataset in [31]. The magnitudes of the modelled and observed extents are very different, with the modelled wMIZ extent being generally twice as large as the observed wMIZ extent in winter. This is a consequence of the modelled wMIZ being a continuous area, while the wMIZ in the IS-2 dataset is more discrete, due to the non-continuous sparse coverage of the altimeter. This difference in magnitudes is even greater when using the 25 km dataset (figure 1*b*), as it contains more cells with fewer than 1000 segments per month. Note that in our analysis, we only sample model cells where IS-2 data are available, the problem being that wave observations by IS-2 are often scattered points between cells with no (or not enough) detected waves. The reasons behind this behaviour are discussed later in this section. Trying to extrapolate a continuous wMIZ from IS-2 observations is not straightforward given the difficulty in assessing uncertainties when computing the WAF. However, it is encouraging that the modelled wMIZ generally shows the same key features as the observed one: a decrease from winter to spring, a sharp rise in the autumn, and a rather stable extent in winter. Using the 90th percentile of $H_{s}$ (in blue) gives a similar evolution of the wMIZ extent overall, but the wMIZ extent is smaller by 20% than when using the monthly maximum of $H_{s}$. The results also show little sensitivity to the $H_{s}$ threshold value in the 1–2 m range, which gives us confidence in the robustness of our wMIZ definition. Assuming IS-2 can detect waves with an $H_{s}$ down to 50 cm highly increases the wMIZ extent, by 50% in winter and a lot more in spring and summer. It also enhances the month-to-month variability of the wMIZ extent, showing for instance a secondary peak after April 2019 similar to the observed wMIZ extent. Thus, the upper bound of the wMIZ extent seems to capture part of the observed wMIZ variability.

We next investigate some quantitative metrics for the modelled wMIZ extent. Define $A_{10}$ as the area that belongs to the modelled wMIZ but not to the observed one, $A_{01}$ as the area that belongs to the observed wMIZ but not to the modelled one, and $A_{11}$ as the area where the model and observation agree that a given location belongs to the wMIZ. A classical way of assessing the ability of a model to capture an extent is to use $A_{11}$ and the errors $A_{10}$ and $A_{01}$ to assign a model score [45]. In our case, we are challenged by the fact that an absence of detected waves is not necessarily indicative of a region without waves, particularly if an area has few wave events in each month. Thus, the IS-2 dataset can provide a large number of false negatives, and a low WAF value (i.e. no waves detected) is less predictive of wave state than a high WAF value. This is visible in figures 1*a* and 2, where areas with a high density of detected waves (WAF > 0.1) are interspersed with missing values and locations with WAF = 0.

A consequence is that the $A_{10}$ mismatch area can be significantly larger than the matching area $A_{11}$, as the modelled wMIZ extent necessarily encompasses a large number of missing values or locations with no detected waves (figure 1*a*). This is illustrated in figure 3*b*, which shows the dependence of $A_{11}$, $A_{10}$ and $A_{01}$, along with the wMIZ extent, on the threshold value applied to $H_{s}$ to define the wMIZ. We can see that $A_{10}$ follows similar behaviour to the wMIZ extent, being much larger than $A_{11}$. To reduce the number of false negatives leading to a large $A_{10}$, we test the effect of increasing the level of confidence in the ‘no waves’ observations by only considering cells with a WAF of more than 30 000 segments (instead of 1000). This reduces the coverage by about 66%, as seen in figure 1*c*, but allows us to define a more conservative version of $A_{10}$, which we call ${\hat{A}}_{10}$. We see that ${\hat{A}}_{10}$ is of the same order of magnitude as $A_{11}$ (figure 3*b*) and increases dramatically when the wMIZ extent becomes very large. This is because as the wMIZ extent keeps increasing, it ends up encompassing large areas of pack ice with very few waves observed. The area ${\hat{A}}_{10}$ can therefore be useful for investigating potential overestimation of the wMIZ extent in a simulation.

The monotonic increase of $A_{01}$ in figure 3*a* illustrates a second challenge in quantitative evaluation of the wMIZ extent. It is due to the high values of WAF that are not captured by the model in the central Arctic pack ice, particularly from May to November [31], visible in figure 2*b*,*c*. It is unlikely that the model in its current configuration would be able to capture these waves, whether these observations are due to noise (false positives) or to wave generation in openings in pack ice [27]. This is because (i) the prediction of the exact location of such openings without assimilating sea-ice deformation is not possible owing to the chaotic nature of the crack formation process [46]; and (ii) the model resolution is likely too coarse to properly resolve wave growth in leads. Indeed, in WW3, wave generation in partially ice-covered cells is scaled by the concentration, but at 25 km resolution, the drop in sea-ice concentration due to divergence is not large enough (generally less than 0.1) to allow for noticeable wave growth in the pack, even though recent observations suggest that this may happen in the Beaufort Sea [27].

We propose separate evaluations of the ability of the model to capture the areas with observed waves, so as not to extend the wMIZ into areas where the IS-2 dataset is confident that there are no waves. First, we compute the fraction of observations captured by the modelled wMIZ by computing the evolution of $S_{01}=A_{11}/(A_{11}+A_{01})$ for REF (figure 3*c*). This metric only samples points with wave observations in the IS-2 dataset and checks whether they are captured by the modelled wMIZ. The model shows a clear seasonal pattern, performing well in the winter and less well in the summer. From December to March, we find that the modelled wMIZ captures about 70% of observations, with little dependence (about 10% maximum) on the definition of the wMIZ. This result varies little with the choice of the $H_{s}$ threshold (±50 cm). If we only account for observations of the WAF with values over 0.25 (dotted lines), where we are highly confident that there are waves, this score is as high as 90% in the 2019 winter (80% using the maximum value or 90th percentile of $H_{s}$). This percentage is lower in 2020 but remains around 80%. This gives us confidence that the model does not underestimate wave activity in the winter, at least in the vicinity of the ice edge, as is visible in figure 2*a*. The model’s ability to capture observations drops from April to August (figure 2*c*), but increases again from September onwards. The results in summer are sensitive to the use of either the maximum value or the 90th percentile of $H_{s}$ for each month (from 0 to 20% of observations captured by the wMIZ), which highlights the stronger sensitivity of the modelled wMIZ to the factors affecting $H_{s}$ magnitude in this season. The reasons behind this underestimation of the wave height in ice from spring to autumn are investigated further in §3c. The main limitation of $S_{01}$ is that it does not inform us whether the model overestimates the wMIZ. For example, a modelled wMIZ encompassing the whole Arctic would include all cells with observed waves, hence getting the perfect score $S_{01}$ = 1. The second step of our evaluation should therefore aim to estimate potential overestimations of the modelled wMIZ. We suggest using $S_{10}=A_{11}/({\hat{A}}_{10}+A_{11})$, presented for REF in figure 3*d*. The higher $S_{10}$ is, the lower the proportion of observations of ‘no waves’ in the modelled wMIZ relative to the number of cells with observed waves within the modelled wMIZ. Changing the number of segments to filter out cells when computing ${\hat{A}}_{10}$ has some effect on the magnitude of $S_{10}$ but little on its qualitative evolution for numbers above 10 000 segments per month (also visible in figure 3*d*). This means that to evaluate different simulations, one can compare the values of $S_{10}$ obtained for each of them, but their interpretation (i.e. whether the modelled wMIZ extent is overestimated or not) requires a qualitative assessment first (as made with figure 2 for REF). Here, we analyse the evolution of $S_{10}$ knowing the qualitative evolution of the wMIZ extent from figure 2, and values of $S_{10}$ using the same methodology applied to other simulations can inform us of the behaviour of these simulations compared to REF (see §3c). We find that $S_{10}$ varies between 0.6 and 1 and shows a seasonal cycle opposite to $S_{01}$ (figure 3*d*). In winter, where the wMIZ extent seems reasonable in figure 2*a*, $S_{10}$ is found to be around 0.7. Therefore, we estimate that a value of $S_{10}$ in the range of 0.6–0.7 means that the modelled wMIZ extent is reasonable, and lower values would mean that the wMIZ extent likely is overestimated. Given that the winter sea-ice extent in the model tends to be overestimated, particularly in the Greenland Sea, a good wave attenuation in the model should lead to a rather underestimated wMIZ extent. In summer, $S_{10}$ reaches values above 0.9, as the model seems to underestimate the wMIZ extent rather than overestimate it (figure 2*b*). In general, the model is not too sensitive to the choice of the $H_{s}$ threshold, and using a more conservative wMIZ extent by taking the 90th percentile is found to increase $S_{10}$ by about 0.1 (15% better than REF). Overall, none of these choices has a significant impact on our results.

### (c) Sensitivity of the modelled wMIZ to model parameters

**Figure 4.**
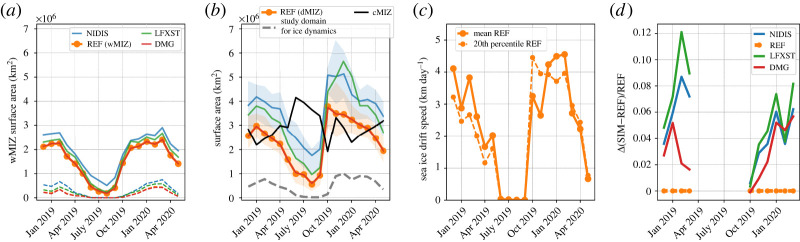
(*a*) Evolution of the wMIZ extent (solid lines) for the different simulations of the sensitivity study. Dashed lines represent the part of the wMIZ extent with sea-ice concentration (ignoring newly formed ice) above 0.8. (*b*) Solid lines are similar to (*a*) but for the dMIZ extent evolution, defined as the area with $D_{\max}$ lower than 100 m on average for each month. The orange and blue shaded areas represent the sensitivity of the dMIZ to the floe size threshold between 30 m and 300 m for REF and NIDIS, respectively. The solid black line represents the cMIZ extent evolution when ignoring the newly formed ice category [38]. The grey dashed line represents the evolution of the area used for the estimation of wave impact on ice dynamics (a monthly averaged $D_{\max}\leq 100\;m$ and sea-ice concentration higher than 0.8 when ignoring newly formed ice). (*c*) Evolution of the monthly average (solid dotted line) and 20th percentile (dashed dotted line) of the ice drift velocity in this area. (*d*) Difference in the 20th percentile of the ice drift velocity in the DMG, LFXST and NIDIS simulations relative to REF in this area. (Online version in colour.)

To investigate what the important factors are in simulating the wMIZ, we compute the quantities $S_{01}$ and $S_{10}$ for the different sensitivity simulations described in §2b. The results are compiled in table 2, and the wMIZ extent for each simulation is shown in figure 4*a*. We also use this sensitivity experiment to investigate the reasons behind the underestimation of the autumn wMIZ extent in REF. This underestimation could be due to false positives in the observations of waves by IS-2. This is likely to be the case in summer (June–August), as the number of segments per cell tends to be lower and melt ponds could be mistaken for ocean surface points when computing the WAF [31]. Because of this high uncertainty in the summer data, we ignore the period June–August in our analysis and focus on September–November (autumn). For this period, moorings measurements support the idea that waves around 1 m high can be found far in pack ice in the Beaufort Sea [27], giving some credit to the high WAF values retrieved from IS-2 in this area.

**Table 2.** Values of $S_{01}$ and $S_{10}$ for the different sensitivity experiments and for the winter period (December to March, 2018–2019 and 2019–2020), spring (April to May, 2019 and 2020) and autumn (September to November 2019). Values in brackets in the $S_{01}$ column correspond to the score accounting only for locations with WAF values above 0.25. Values in brackets in the $S_{10}$ column correspond to the score using a monthly maximum $H_{s}\text{ of }0.5\;m$ to define the upper bound of the modelled wMIZ extent. The period from June to August is not considered here because of the likely high level of noise in the IS-2 observations.

	December–March		April–May		September–November	
simulation	$S_{01}$	$S_{10}$	$S_{01}$	$S_{10}$	$S_{01}$	$S_{10}$
REF	0.70 (0.80)	0.73 (0.63)	0.39 (0.50)	0.86 (0.72)	0.41 (0.50)	0.83 (0.74)
DMG	0.70 (0.80)	0.73 (0.63)	0.39 (0.50)	0.86 (0.73)	0.40 (0.50)	0.84 (0.73)
LFXST	0.72 (0.82)	0.67 (0.51)	0.43 (0.54)	0.83 (0.66)	0.44 (0.53)	0.79 (0.69)
NIDIS	0.73 (0.83)	0.58 (0.39)	0.45 (0.56)	0.77 (0.55)	0.51 (0.62)	0.80 (0.60)

We first look at DMG to check that activating the link between damage and fragmentation has little impact on the wMIZ extent. This is needed as this link is activated in other simulations investigating the sensitivity of other parameters compared to REF (table 2). The impacts of activating this link are described in detail in [23] and could affect wave attenuation by making the ice thicker in the wMIZ after a long period, but we find that differences between DMG and REF are negligible for both the extent metrics, $S_{10}$ and $S_{01}$.

**Figure 5.**
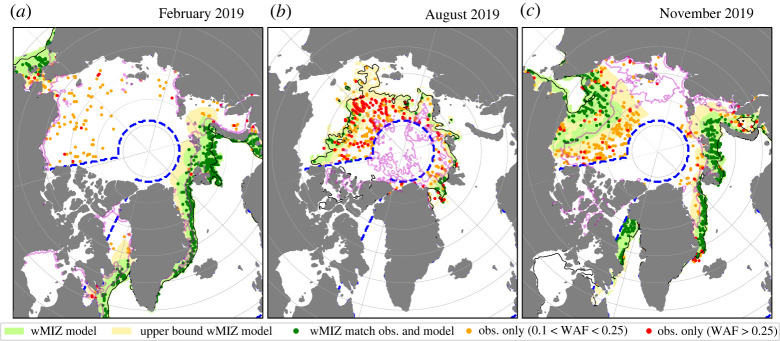
Same as figure 2 but for the NIDIS simulation. (Online version in colour.)

The NIDIS simulation changes the attenuation process by removing inelastic dissipation, which is efficient at attenuating waves with longer wave periods (greater than 10 s) and in broken ice [15]. Inelastic dissipation assumes that sea ice is a viscoelastic solid flexing repeatedly under the wave action, which dissipates energy. Its effect vanishes when the floe size becomes smaller than the wavelength of the waves [15]. To compensate for this loss of attenuation compared to REF, we increase turbulent friction by increasing the roughness length (table 1; see [39]). In winter, NIDIS does slightly better than REF at capturing cells with observed waves ($S_{01}=0.73$ vs 0.70 in REF), but at the cost of overestimating the wMIZ extent, with a value of $S_{10}$ dropping below 0.6, which is approximately 20% less than $S_{10}$ for REF. The wMIZ extent increases by about 15% compared to REF (figure 4*a*). This increase of the wMIZ extent in NIDIS results in an increase of $S_{10}$, as the additional wMIZ area compared to REF mostly encompasses regions with few waves observed by IS-2 and hence most likely overestimates $H_{s}$ in these locations (figure 5*a*). Moreover, this increase of the wMIZ mostly corresponds to areas in the Greenland and Barents Seas, where we would expect an underestimation of the wMIZ given that the model consistently overestimates the ice extent in these regions. This increase is particularly visible when considering the upper bound of the wMIZ extent (using the threshold $H_{s}=0.5\;m$). Using this definition of the wMIZ extent, $S_{10}$ decreases by 35% between REF and NIDIS. In autumn, however, NIDIS captures more of the waves detected in the Beaufort and Chukchi Seas (figure 5*c*), and the wMIZ extent does not appear to be overestimated, at least when using $H_{s}=1.5\;m$ as a threshold. This is visible in the $S_{10}$ values, which are roughly similar for REF and NIDIS, while $S_{01}$ in NIDIS is equal to 0.51, 20% higher than in REF (0.41). The comparison between REF and NIDIS suggests that inelastic dissipation may not be a dominant mechanism in autumn, when sea ice in the MIZ is forming and consolidating. In winter, however, inelastic dissipation, or in general an attenuation process efficient at attenuating long waves in unbroken ice, seems necessary to obtain enough wave attenuation in thick pack ice, such as north of the Barents and Greenland Seas. This is particularly visible as few wave observations are found beyond the contour of sea-ice concentration higher than 0.8 in February (figure 2*a*).

The overestimation of the wave attenuation from inelastic dissipation in REF in autumn could also originate from an overestimation of the floe size. This is likely to be the case in our model, as we ignore the effects of processes other than waves [30] and assume that ice forms as an unbroken, continuous, thin ice sheet [23]. Lowering the flexural strength (LFXST) tends to increase the wMIZ extent, as it reduces the wave attenuation by making it easier for waves to break the ice, reducing the amount of inelastic dissipation in the model. The value of the flexural strength in REF was taken from [21], where it was shown to give approximately the right extent of broken ice after a wave event in the Beaufort Sea, while the value in LFXST is the one used in a number of other studies and originally suggested in [12]. Changes in $S_{10}$ and $S_{01}$ in winter and autumn are relatively small, which means that lowering the flexural strength has little impact on the wMIZ extent. However, if we consider the upper bound of the winter wMIZ, we find a relative decrease of $S_{10}$ by 15% between REF and LFXST, suggesting that waves with lower heights are able to propagate significantly farther when the flexural strength is reduced. This suggests that the flexural strength value used in [21] (taken in the upper range of what is known of the flexural strength of sea ice) and in REF gives a better wMIZ extent overall, at least for a conservative approach.

As neither the inelastic dissipation nor the flexural strength fully explains this underestimation of the autumn wMIZ, we look more closely at the sea-ice properties. The sea-ice evaluation (§3a and table S2 in the electronic supplementary material) shows that the autumn sea-ice extent in the Beaufort and Chukchi Seas is slightly overestimated, but also that sea-ice concentration shows a clear positive bias (figure S2, electronic supplementary material), which likely explains part of the underestimated wMIZ extent. This is supported by the finding [27] that waves measured in pack ice are mostly locally generated, which requires the presence of open water that the model seems not to capture. However, this overestimation of sea-ice concentration could be partly compensated for by underestimation of the ice thickness at the end of summer, which should have the opposite effect. Adjusting the modelled ice extent and thickness in a specific region is not straightforward, particularly in a simulation with prescribed oceanic and atmospheric conditions. The best way to address this issue would be to use data assimilation, as in [41], but this is beyond the scope of the present study. Note that we also investigated the sensitivity of our simulation to the wind speed by increasing $\beta_{\max}$, a non-dimensional wave growth parameter that controls the input of energy from the wind to the waves, but found very little impact within a reasonable range of values.

### (d) Comparison with a floe size-defined MIZ

We now investigate how the wMIZ definition would compare with a floe size-based definition. Such definitions of the MIZ have been suggested in other studies [13,14,23], generally using the maximum floe size $D_{\max}$ as a metric. Using the same coupled system as here, Boutin *et al*. [23] investigated the impact of waves on sea-ice dynamics in a MIZ defined as the area with a $D_{\max}$ lower than 100 m. This maximum floe size is defined in neXtSIM as the 90th percentile of the areal floe size distribution, and mostly depends on the wavelength of the incoming waves [23]. It varies between 10 m (broken thin ice) and 1000 m (unbroken ice).

Here, we use the same definition as in [23], using the area where the monthly averaged value of the maximum floe size ($D_{\max}$) is lower than 100 m, and refer to this area as the dMIZ. We also look at the sensitivity of this definition in the 30–300 m range; 30 m corresponds roughly to the smallest floe size that can undergo flexural failure for an ice thickness less than 2 m [47], while 300 m corresponds to the maximum floe size associated with long waves (with periods of around 14 s) and is an upper bound for the dMIZ used in different studies (e.g. [22]). For all the simulations, the dMIZ extent is very similar to the wMIZ extent investigated before (figure 4*b*), with the exception of the months of September to November, where the area of broken ice exceeds the wMIZ extent by about 33% for REF. This is similar to the observed wMIZ, which also shows a clear peak in October. This similarity is not unexpected, as floe size in the current set-up is mostly influenced by wave break-up, and it is likely that more differences would emerge if other processes were also affecting the floe size. The dMIZ shows a rather high sensitivity to the choice of the value of $D_{\max}$, particularly in the range of 30–100 m, even if the qualitative evolution of the dMIZ extent remains unchanged. The dMIZ also shows greater sensitivity to the flexural strength wave attenuation (NIDIS vs REF) than the wMIZ, particularly in the autumn. This greater sensitivity of the dMIZ to the flexural strength is quite logical, as this latter quantity is used to determine whether break-up occurs [14,15]. We have some confidence in our fragmentation criterion (described in [15]), as Voermans *et al*. [48] note that it is consistent with observations. From our evaluation of the wMIZ, where REF performed better than LFXST, we suggest that the dMIZ in REF is a more reasonable estimate than LFXST.

The greater sensitivity of the dMIZ to wave attenuation processes than the wMIZ (when looking at REF and NIDIS) is possible because the wMIZ extent is constrained by the minimum $H_{s}$ detected by IS-2, but waves with $H_{s}≃0.3\;m$ can still break the ice [21], and observations do not allow us to constrain the attenuation for these waves. This is a key limitation of using the simple wave-detection approach to IS-2 data when it comes to constraining the modelled wave impact on ice in a model.

While we do not expect the model to predict $D_{\max}$ precisely, as the floe size distribution in the model is poorly constrained because of the limited observations available, it is still possible to interpret the meaning of average $D_{\max}$ values. The area defined by $D_{\max}$ lower than 300 m roughly corresponds to the area where the minimum value of $D_{\max}$ is lower than 100 m (figures S4 and S5, electronic supplementary material), meaning that fragmentation has occurred at least once in the month. It gives an upper bound on the ice-covered area impacted by waves, which is larger than the wMIZ extent, particularly in autumn. The area where $D_{\max}$ is lower than 100 m on average over the month corresponds to regions that are regularly undergoing wave-induced fragmentation, while the MIZ extent defined using $D_{\max}$ lower than 30 m is very similar to the wMIZ defined using the 90th percentile of $H_{s}$ for each month (figure 3*a*).

We also compare the wMIZ and the dMIZ with the classical definition of the MIZ based on the concentration (cMIZ), i.e. the area with a sea-ice concentration between 0.15 and 0.8 [49,50] (figure 4*b*), which is a useful metric for climate model evaluation [51]. Here, we take the upper bound of the cMIZ to be 0.8, ignoring the fraction occupied by the ‘newly formed ice’ category [38]. This ice type is meant to represent thin forming ice (frazil, nylas etc.), which may not be captured by satellite observations [52]. More importantly, this not-consolidated ice has no internal stress and its strength is therefore not affected by wave-induced ice break-up in the model [23]. As noted in [31], the wMIZ in REF mostly overlaps with the cMIZ (figure 2*a*,*c*), except in limited areas where the wMIZ encompasses more compact ice with a concentration higher than 0.8, mostly in the Greenland Sea and north of the Barents Sea. It is only when the wMIZ (and hence the dMIZ) extent is overestimated in winter (in NIDIS) that these areas of pack ice affected by waves become really significant (up to 25% of the wMIZ, instead of around 15% maximum in REF).

### (e) Impact on sea-ice dynamics

One of the main goals of waves-in-ice modelling is to assess the potential of waves to affect sea-ice evolution, as was done with the model presented here and the sea-ice dynamics in [23], the latter focusing only on the Barents Sea in October 2015. The impact on ice deformation and drift compared to a reference uncoupled simulation was found to be significant in the aftermath of storm events, as areas of compact ice that had been fragmented were more mobile due to lower ice strength. However, on average over the whole 40-day period, the impact on the average drift was small (7%) and limited to a small region.

Here, we compare the simulations DMG, LFXST and NIDIS, in which fragmentation lowers the ice strength of compact ice, with REF (figure 4*d*). For a fair comparison between these four simulations, we define a fixed geographical area for each month that is independent of the simulation. We take it to be the area where $D_{\max}$ in REF is lower than 300 m and the monthly average concentration is higher than 0.8 (ignoring the forming ice as defined in [38], as it has no ice strength), as it gives a reasonable estimate of where fragmentation happens at least once during the month. This area is small, representing about one-third of the wMIZ extent in winter and quickly dropping to almost zero between April and October/November (figure 4*b*). We therefore limit our analysis to the months from October to March.

We find little difference in the average sea-ice drift in the selected area between the different simulations (not shown). A greater difference is visible when looking at the 20th percentile of drift speed, which is of the same order of magnitude as the average drift speed. This is consistent with [23], i.e. the impact was mostly an increase in mobility of fragmented ice when the drift is slow. The greatest increase in the drift speed for this percentile is found for LFXST, with drift velocities 12% higher than in REF, likely because a lower ice strength increases the amount of ice break-up. However, the increase in ice drift does not exceed 6% in DMG, where the wave attenuation and ice break-up are the same as in REF. Overall, the impact is therefore not significant on the sea-ice evolution in the model. This does not contradict [23]: waves can affect sea-ice mobility, but their impact remains limited in time and space. The fact that we still find an increase in low drift speed means that the updated neXtSIM rheology [33] used here compared to [23], with more resistance to convergence, does not prevent waves from affecting sea-ice deformation.

## 4. Discussion and conclusion

We have compared the wMIZ extent in a coupled wave–sea-ice model with observations from IS-2, showing satisfying results for our reference simulation. We have suggested two quantitative metrics, $S_{01}$ and $S_{10}$, for assessing whether the modelled wMIZ extent is underestimated or overestimated. These metrics, $S_{10}$ in particular, have to be considered in combination with a qualitative assessment of the wMIZ. Using our methodology, values of $S_{10}$ above 0.66, such as for REF in winter, mean that the model does not overestimate the extent. Our method of comparing a wave–ice model with WAF observations provides a simple but robust way to evaluate model performance over a full year and for the whole Arctic. However, quantitative interpretation of the WAF remains largely uncertain and limits its use to assessing whether a modelled wMIZ is underestimated or overestimated. This is useful for investigating wave impact on sea ice in coupled models but limited when it comes to understanding wave attenuation, for instance. The retrieval of wave spectra information, currently in development [36], should make comparison with other types of data easier [37]. Combining IS-2 data with remote sensing of floe size [30] and analysis of mooring measurements [27] in ice could also contribute to increasing our understanding of the WAF and wave–ice interactions in general.

Our results suggest the need for a modelled mechanism to dissipate wave energy in compact, thick, solid ice. Sea-ice models including floe size distributions should therefore be careful to have strong wave attenuation in the winter so as to not strongly overestimate the impact of waves in this season. A consequence of strong attenuation in pack ice in winter is that even when the wMIZ extent peaks due to large open-ocean waves, the amount of thick, compact ice affected by waves in the Arctic remains small. This is likely not the case in the Southern Ocean, where large wave events and long-period waves are much more frequent [19,36]. This has recently been shown in [36], wherewaves in IS-2 data were detected far beyond the in-ice extent of the cMIZ.

A consequence for sea-ice dynamics is that taking wave-induced fragmentation into account when computing the ice strength differs little from using classical concentration-based parametrizations [53]. However, in [23] Boutin *et al*. assumed that the recovery time scale of ice strength after a fragmentation event is the same as for brittle fracture in the pack ice, but wave break-up is likely to create a larger number of cracks in the sea ice and may have a longer-lasting effect. Another limitation of their approach is that it neglects the impact of floe–floe interactions on internal stress, which can be significant [54].

## Data Availability

The ICESat-2 dataset is publicly available at https://doi.org/10.1594/PANGAEA.918199. Scripts and modified routines will be available on GitHub after publication of this paper. The processed model outputs are available at https://ige-meom-opendap.univ-grenoble-alpes.fr/thredds/catalog/meomopendap/extract/SASIP/model-outputs/WW3-neXtSIM/catalog.html. The CS2SMOS sea-ice thickness product is available at ftp://ftp.awi.de/sea_ice/product/ (last accessed January 2022). The low-resolution daily sea-ice drift product and sea-ice concentration products from OSI-SAF can be found at ftp://osisaf.met.no/archive/ice/ (last accessed January 2022). The data are provided in the electronic supplementary material [55].
